# Interspecific competition among aphid parasitoids: molecular approaches reveal preferential exploitation of parasitized hosts

**DOI:** 10.1038/s41598-019-56187-3

**Published:** 2019-12-23

**Authors:** Sebastián Ortiz-Martínez, Jean-Sébastien Pierre, Joan van Baaren, Cécile Le Lann, Francisca Zepeda-Paulo, Blas Lavandero

**Affiliations:** 1 Universidad de Talca, Instituto de Ciencias Biológicas, Laboratorio de Control Biológico, Avda. Lircay s/n, Talca, Chile; 2 Université Rennes (Univ Rennes), UMR-CNRS 6553, ECOBIO, F-35042 Rennes, France

**Keywords:** Community ecology, Molecular ecology

## Abstract

When a guild of species exploit the same limited resources, interspecific competition induces the exclusion of inferior competitors, in which case, interspecific recognition mechanisms are needed. Here, we address resource partitioning and interspecific competition among three main solitary parasitoid species attacking the same host resource, the aphid *Sitobion avenae* in wheat fields. Optimal host acceptance models predict that parasitoid species should prefer attacking unparasitized hosts when they are available in order to maximize their fitness, as already parasitized hosts are less valuable for laying eggs, especially for inferior competitors. Therefore, we expected the level of competition (multiparasitism) in the field to increase at low host density. By using a combination of taxonomical (determination) and molecular (PCR-based) approaches, we assessed the species of all parasitoid adults and immature stages within aphid hosts. Our results demonstrate that, early in the season, the multiparasitism rates were low, whereas they were high in the mid-late season, corresponding to an aphid density decrease over time. Moreover, parasitoid species could not have been exploiting host resources randomly and the better competitor, *Aphidius ervi*, seemed to be foraging preferentially on hosts already parasitized by the inferior competitor *A*. *rhopalosiphi*, even when unparasitized hosts were still available. This could be due to differences in their host detection capability, as species with a narrow host range may be better at detecting their hosts in comparison with species with a greater host range, such as *A*. *ervi*, with a greater host range within the guild. Our study suggests differences in the host exploitation of two prevalent parasitoid species through the main period of aphid colonization, which still allowed the coexistence of a third inferior competitor (*A*. *rhopalosiphi*) within the assemblage, in spite of some negative interactions (multiparasitism) and redundancies.

## Introduction

When sympatric species within natural communities are using limited resources, they can directly or indirectly exclude their competitors, or avoid competition through resource-use complementarity and niche differentiation^1–5^. Resource partitioning can promote species’ coexistence within a guild via qualitative, temporal or spatial differences in how resources are exploited^6–8^. For instance, species may differ in their strategies or abilities to find and exploit resources, and these differences are often linked to a generalist or specialist diet^9^.

It has been propounded that predator species with a wider diet breadth may have a reduced competitive ability per resource type. However, by having an advantageous set of alternative resources, they may be less affected than specialists when specific food items are not available in the environment at a given time^10^. On the other hand, predator species with a narrower diet breadth tend to specialize in a specific food-resource, achieving better fitness^11,12^. Indeed, specialized predator species are often better at overcoming prey defences (chemical and behavioural) and excel in resource finding. Nevertheless, they could also be more affected by variations in the abundance and distribution of their main prey^13^.

To address questions about the coexistence of species with varying degrees of diet breadth, parasitoids are model organisms. They have an intimate relationship with their insect hosts, as they complete their development inside or attached to them. Accordingly, parasitoids depend strongly on the abundance and distribution of their hosts, which greatly influences their reproductive opportunities^14,15^. In parasitoids, specialism and generalism are defined in terms of phylogenetic conservatism consumption (e.g., attacking hosts in the same family)^16^. The interaction between hosts and parasitoids often promotes host specialization and this has been observed mainly through an increase in host-use efficiency^17^. Furthermore, parasitoids with smaller host ranges will find their hosts more easily compared to parasitoids with greater host ranges^18^. Assemblages of parasitoid species with different host ranges could allow some resource sharing and consequently the coexistence of parasitoid species in a certain space and time^15^. In particular, parasitoids with a narrow host range exhibit greater host-use efficiency and/or can overcome host defences more easily, as they specialize in hosts with similar traits^19^. On the other hand, even parasitoids with a wider host range may still adjust their foraging strategies to their main host species, which could reduce their efficiency regarding finding and exploiting other hosts^17^.

Even after being parasitized, a host can still be used by conspecifics (superparasitism) or by other parasitoid species (multiparasitism), but in solitary parasitoids, only one individual emerges from the host. In this case, the optimal foraging theory (OFT) predicts that a parasitoid will optimize its fitness by parasitizing the best suitable resource or the most accessible one, rather than using any available random resources^20^. Therefore, most parasitoid species have evolved the ability to identify parasitized hosts to avoid larval competition^14^. Interspecific host discrimination is less frequent than intraspecific host discrimination^21,22^ and has been shown in two particular situations: first, in the case of an inferior competitor identifying its superior competitor and second, when competitors are phylogenetically closely related species. In the latter case, the signals used for intraspecific host discrimination in each species could be similar and recognized interspecifically^22^. Nevertheless, the OFT also predicts that already parasitized hosts will be accepted when oviposition resources are scarce^23,24^, which may favour species with a wide diet breadth and/or superior competitors. By contrast, when hosts are present at high densities, parasitism rates may decrease, due to a dilution effect of the resource and/or their own egg limitation, which may also lead to reduced (multi)parasitism rates^25–27^.

The aphid-parasitoid system is particularly interesting, as the main parasitoid species belong to one genus, *Aphidius* (Hymenoptera: Braconidae) and some of them present different diet breadths. For instance, the aphid parasitoid *Aphidius ervi* Haliday is considered a habitat/host generalist species, which attacks several aphid species on different plant families, but mainly *Acyrtosiphum pisum* Harris on legumes^28–30^. By contrast, the species *Aphidius rhopalosiphi* De Stephani-Perez and *Aphidius uzbekistanicus* Luzhetzki exhibit a narrower host range and are considered to be habitat specialists foraging on cereal aphids, mainly *Sitobion avenae* Fabricius^28–30^. In terms of competitive abilities, *A*. *ervi* is known to win the competition against *A*. *rhopalosiphi*, as it often lays two or more eggs in the same aphid in contrast to other *Aphidius* species^30,31^. *A*. *rhopalosiphi* is known to be affected by the defensive behaviour of *S*. *avenae* and as soon as aphids emit defensive signals, this parasitoid leaves the host patch partially exploited. Conversely, *A*. *ervi*, which is not affected by the defensive behaviours of *S*. *avenae*, tends to fully exploit host patches^22,30,32^. Both species have intraspecific host discrimination abilities and generally avoid hosts parasitized by females of their own species^33,34^. Moreover, both species are also capable of discriminating unparasitized *S*. *avenae* aphids from aphids parasitized by other species and more often accept those which are unparasitized^31–33,35^. Behavioural manipulation experiments have shown that through external cues (by using antennal perception), the recognition of unparasitized and parasitized hosts occurs before the oviposition^32^. In this situation, this species possesses the ability to measure resource value (i.e., host quality), important in parasitoid foraging strategy studies. Moreover, for *A*. *rhopalosiphi* and other *Aphidius* species, the discrimination of unparasitized and parasitized hosts by other species can also occur after ovipositor insertion, indicating that an internal marker of parasitism can be also be used^33^.

In Central Chile, the English grain aphid *S*. *avenae* is the most abundant aphid in cereal crops, as it constitutes more than 85% of the total aphid abundance^36^. The main natural enemies responsible for the regulation of cereal aphids are mainly a group of native and introduced coccinellid species, as well as several introduced parasitoid species^36–38^, which have been shown to partition the aphid populations depending on the landscape context^37,38^. An assemblage of eight parasitoid species co-occur in wheat fields^37,39,40^, with *A*. *ervi* comprising the most common species in cereal crops with the greatest host range (15 host species in different crops) and a high prevalence in Chile (more than 38% of the emerged adults from *S*. *avenae* hosts)^40^. Although *A*. *rhopalosiphi* and *A*. *uzbekistanicus* (habitat specialists) are present in this system, they have a smaller host range (six and three host species in total, and 12% and 28% of the emerging adults parasitizing *S*. *avenae* in cereal fields, respectively)^39,40^. Despite previous studies suggesting that the biological control of cereal aphids in Central Chile has an important effect on pest suppression, negative interactions among coccinellid predators and between predators and parasitoids are common^38^. Negative interactions, such as interspecific competition for aphid hosts, are likely to occur in this system, which may also produce redundancies in biological control. In this study, we addressed the competition between these three main *Aphidius* parasitoid species attacking *S*. *avenae* in wheat fields in Central Chile. Molecular approaches have been extensively used to understand trophic ecology at the field scale during recent years^41–44^ allowing us to determine whether each aphid host collected in the field was (multi)parasitized and the identity of the parasitoid species. On the other hand, by identifying adult parasitoids emerging from their hosts sampled in the field and reared in the laboratory, we were able to determine the surviving species (i.e., the outcome of the competitive interaction) and their prevalence in the field. According to the OFT, we hypothesized that multiparasitism is highly frequent in the field when aphid resources are scarce and less frequent when the aphid population abundance is high. Furthermore, as *Aphidius* species differ in their competitive abilities, we expected to find higher abundances of the best competitor when resources are scarce, but not necessarily when aphids are abundant. Therefore, we expected differences between both methods of species identification (molecular and rearing), with the superior competitive species more abundant among the emerging adults than those detected as immature stages within the hosts (through the molecular approach). The reverse situation may be observed for the species with lower competitive abilities. Finally, we expected all parasitoids to avoid laying eggs in hosts already parasitized by other species and that oviposition events should not be performed randomly on the available hosts, with single rather than multiple ovipositions being more frequent.

In order to test these hypotheses, the abundance of *S*. *avenae* was estimated over the season, as well as the multiparasitism rates, the effective oviposition frequencies for all parasitoid species and the relative abundance of each parasitoid species emerging from the parasitized hosts.

## Materials and Methods

### Sampling

Live apterous aphids were sampled during the main period of aphid colonization (16^th^ October 2013) until its breakdown (10^th^ December 2013). Aphids were sampled from 20 wheat fields in Central Chile, the main wheat production area (for more details see^37^), over five sampling dates separated by 15-day intervals. For each date and field, in order to estimate the aphid density, all living apterous *Sitobion avenae* were sampled (using a small brush) from 20 randomly selected wheat tillers in three different plots of 1 m^2^ per field (60 wheat tillers in total per field) (1,819 individuals in total). Aphid identity was corroborated using taxonomic keys^45^. Inspected plants were chosen from a distance of at least 5 m from the edge of each field in order to avoid border effects. From these collected aphids, a sub-sample of a maximum of 10 individuals (per sampling date and field) was taken and conserved in single 1.5 ml sterile micro-centrifuge tube containing 99% ethanol and then conserved at −20 °C. This sub-sample was kept in order to perform molecular analyses for the diagnosis of endoparasitism occurring at the field level. In order to assess the parasitism status and to determine the emerging parasitoid species, extra aphid sampling was conducted on the first date (16^th^ October, the period of high aphid density at the field) and the fourth date (26^th^ November, a period of low aphid density). All living aphids were picked carefully from 20 randomly selected wheat tillers per field (517 individuals in total) and then reared in the laboratory on oat plants (*Avena sativa* Linneaus) until adult emergence from mummies (i.e., classical rearing approach). Mummies were kept until the emergence of adults and for a maximum of 21 days.

### Parasitoid species identification by the molecular approach

In order to determine if an aphid was parasitized and a parasitoid species was present, a diagnostic multiplex PCR was performed using a combination of species- and genus-specific cytochrome oxidase I mtDNA primer pairs (see Table 2 in^44^). This methodology allowed us to identify all species in a two-step PCR, separating the *Aphidius*-group parasitism in the first step and subsequently the specific detection of the DNA of the *Aphidius* species. Consequently, we identified eight primary parasitoids: *Aphidius ervi*, *A*. *avenae* Haliday, *A*. *rhopalosiphi*, *A*. *uzbekistanicus*, *Ephedrus plagiator* Nees, *Praon gallicum* Starý, *P*. *volucre* Haliday and *Toxares deltiger* Haliday inside each aphid sample. Our modifications to the multiplex PCR protocol were as follows: 0.8 µl of Buffer 5 × (Promega), 0.1 µl of dNTPs (10 mM), 0.8 µl of MgCl2 (Promega) and 0.06 µl Taq polymerase (Promega). The cycling conditions consisted of 15 minutes (min) at 95 °C, 40 cycles of 30 second (s) at 94 °C, 3 min at 62.5 °C, 1 min at 72 °C and a final elongation for 10 min at 72 °C. When samples tested positive with the *Aphidius*-group primer pair (the genus-specific primer pair), four different singleplex PCRs were performed in order to identify the parasitoid at the species level (species-specific primers) for four species: *A*. *avenae*, *A*. *ervi*, *A*. *uzbekistanicus* and *A*. *rhopalosiphi*. For all multiplex and singleplex PCRs, a minimum of three negative controls were included in each batch of 96 analysed samples (double distilled H_2_O substituting DNA extracts) to avoid false negatives. The PCR products were separated by electrophoresis with 3% agarose gels using 100 V for 120 min (multiplex PCR) and 90 min (singleplex PCRs). This method allowed us to detect multiple parasitoid species that oviposited at least once in the same aphid. However, this method did not allow superparasitism to be identified. The number of each DNA parasitoid species detected per sample was summed for each sampling date and field in order to obtain the minimal number of ovipositions as a proxy of the level of multiparasitism of each aphid at the field level. One positive DNA detection for a given parasitoid species indicated at least (therefore ‘minimal’) one individual (egg or larvae) of this given species. This minimal number of ovipositions per date was calculated independently per aphid sample; in the case of the number of ovipositions by a single-species (regardless of the identity), only the samples which were positive for only *one* parasiotid DNA detection were selected and summed. Accordingly, for the number of ovipositions by two, three and four species, only the samples which were positive for *two*, *three* and *four* different parasitoid species DNA detections (respectively) were selected, counted and summed. This method did not allow the detection of several parasitoids eggs/larva of the same species present inside one aphid, so we were not able to estimate the superparasitism rate.

### Taxonomical identification of parasitoid species by the rearing approach

The living aphids taken from the extra sampling (the first and fourth sampling dates) to assess the outcome of the competitive interaction and the identity of the species were transferred to the rearing chambers and treated under controlled laboratory conditions, which allowed the development of aphids and parasitoids (20 °C, 50–60% RH, D16:N8 of photoperiod). Living aphids were placed in 5 cm diameter Petri dishes (maximum 30 individuals per dish) and fed using oat plants. Aphids were kept until adult parasitoid emergence. Emerged adult parasitoids were identified to the species level using taxonomic keys for Aphidiinae parasitoid species^46,47^.

### Statistical analyses

The statistical software R v3.2.3.^48^ was used to analyse the data. To estimate the dynamics of the aphid density over the season, the mean number of aphids per date was analysed using generalized linear mixed models (GLMM)^49^ using the sampling date as a fixed factor and the field as a random factor. A Poisson error distribution was used and there was no overdispersion in the data.

In order to obtain a proxy for the potential number of parasitoid species attacking each aphid at the field level, the minimal number of ovipositions was calculated using the molecular approach explained above, within 641 collected aphids. Then, to compare the observed and expected frequencies, an analysis of the interspecific host discrimination was conducted as follows: A distribution of unparasitized, singly parasitized and multiparasitized aphids (two, three or four different species) was built using a Monte-Carlo simulation, under the null hypothesis of random host acceptance, assuming that no host discrimination ability was present, that a maximum of one egg could be laid by the parasitoid per oviposition and also that all the detected eggs and larvae survived until aphids were collected in the field^33^. Knowing the number of each parasitoid species detected by the molecular analysis for each date, the number of aphids parasitized by one, two, three or four different species were randomly distributed among the set of total available aphids. This process was replicated 10,000 times, ensuring stabilization of the theoretical counts at the second digit after the decimal point. We provide an R script in the Supplementary Material (see Supplementary Material *newcalc*.*R*). Then, this theoretical (random) distribution was compared using an χ^2^ test with the observed distribution of the number of aphids parasitized by one, two, three or four different species.

The relative proportion and the minimum number of ovipositions of the three most prevalent parasitoid species (*A*. *ervi*, *A*. *rhopalosiphi* and *A*. *uzbekistanicus*) were measured for each sampling date, using the molecular detection as well as the proportion of adult parasitoid emergence obtained by the rearing approach. For the rearing approach, the parasitism rate was calculated as the number of adult parasitoid individuals which emerged per species from the reared aphids, with respect to the total number of reared aphids per date and field. For the molecular detection of parasitized aphids, the parasitism rate of each parasitoid species was calculated as the number of aphids which tested positive for parasitoid DNA, with respect to the number of all screened aphids per date and field.

Finally, during the high aphid density period (date one) and for the low aphid density period (date four), for the most prevalent species, both the number of parasitoid DNA detected samples using the molecular approach and the number of emerged individuals (adult parasitoids) using the rearing approach were compared using a t-test with a Bonferroni correction for multiple comparisons under the null hypothesis that each species could be the successful emerging species in a multiparasitism scenario.

### Ethical approval

All applicable international, national, and/or institutional guidelines for the care and use of animals were followed. This article does not contain any studies with human participants performed by any of the authors.

## Results

### Aphid abundance and (multi)parasitism rate

**Figure 1:**
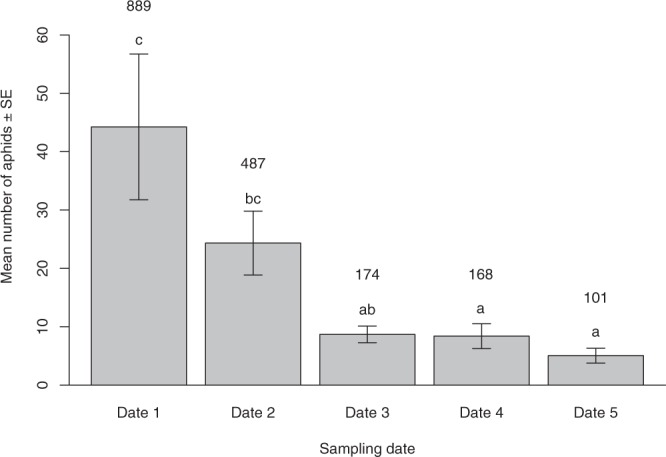
Mean number of *Sitobion avenae* aphids (±standard error (SE)) per each sampling date in 20 fields with 60 wheat tillers per field per each sampling date: date one (16^th^ October), date two (4^th^ November), date three (13^th^ November), date four (26^th^ November) and date five (10^th^ December). Numbers on each column indicate the total aphid abundance per sampling date and different letters indicate statistical differences at *p* < 0.05.

**Figure 2:**
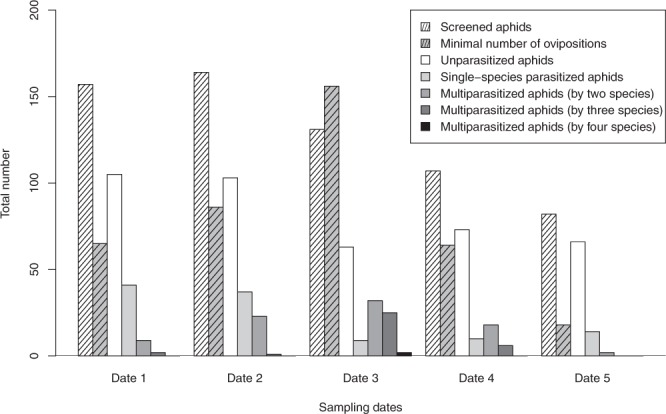
Total number of screened aphids, minimal number of ovipositions, unparasitized aphids and parasitized/multiparasitized aphids per each sampling date using the molecular approach.

Across the entire sampling period (five dates), a total of 1,819 individuals of *Sitobion avenae* were collected in the 20 fields. The mean number of aphids per field differed significantly between dates (χ^2^ = 33.28, *df* = 4, *p* < 0.001) with a higher abundance of aphids on the first date, then decreasing from the second date and lowest on the final sampling dates (dates three, four and five) (Fig. 1). The total number of screened aphids, parasitized aphids and the number of ovipositions are provided in Fig. 2, showing that the competition between parasitoids was the highest on date three.

**Figure 3:**
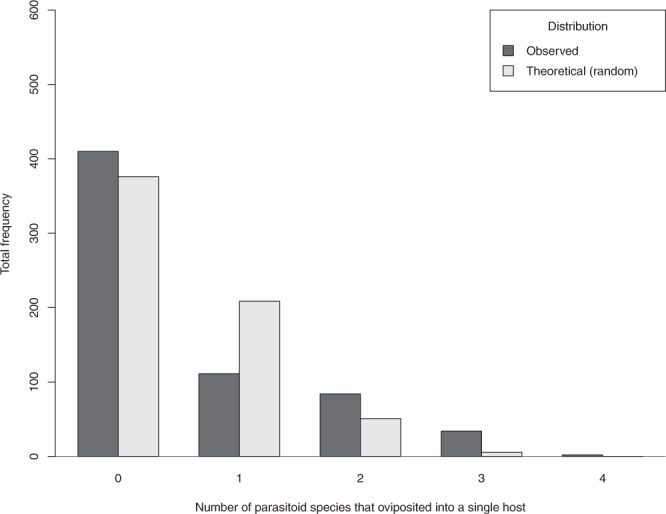
Comparison of the theoretical and the observed distributions of aphids containing zero to four parasitoid species. The theoretical distribution of zero, one, two, three and four parasitoid species in grey was calculated based on the total number of aphids collected during the season (641), parasitized by zero to four parasitoid species under the hypothesis of random host acceptance. The observed distribution of the number of parasitoid species, in black, was obtained by molecular detection of parasitoid DNA in these 641 aphids.

The theoretical frequency distribution of the number of parasitized aphids by different parasitoid species (under the hypothesis of no discrimination ability with a random choice of aphids) differed significantly from the observed number of parasitized aphids determined by the molecular approach (all dates pooled) (χ^2^ = 93.42, *df* = 3, *p* < 0.01) (Fig. 3). There was: (a) a greater number of unparasitized aphids than expected (denoted by “0” parasitoid species in Fig. 3); (b) a lower number of parasitized aphids that contained the DNA of only one parasitoid species (denoted by “1” parasitoid species that oviposited into a single host in Fig. 3); and (c) a greater number of parasitized aphids which contained DNA from more than one parasitoid species (multiparasitism) (denoted with “2”, “3” and “4” parasitoid species that oviposited into a single host in Fig. 3). Taking into account each date separately, the observed number of parasitized aphids by a single species was significantly lower, but the observed number of parasitized aphids by more than one species and unparasitized aphids were significantly higher than predicted by the theoretical random distribution for sampling dates two, three and four (when the competition was high). For date one (higher host density), the contrary was observed. During date five, there were no significant differences between the observed and expected number of unparasitized or (multi)parasitized aphids, however, very few parasitized aphids were found (only 16) (Table 1).

**Table 1:** Comparison of the total number of the observed and theoretical (random acceptation of multiparasitized hosts) distribution of the number of aphids per each sampling date considering the number of aphids parasitized by one, two, three or four different species and unparasitized aphids.

		Distribution of the number of aphids					Total sampled aphids	Total parasitized aphids (%)	χ^2^	p
		Unparasitized	Parasitized by one species	Parasitized by two species	Parasitized by three species	Parasitized by four species				
Date 1	Observed	105 (66.9%)	41 (26.1%)	9 (5.7%)	2 (1.3%)	0 (0%)	157	52 (33.1%)	6.87	0.032^*^
	Theoretical	109.04	42.8	4.98	0.17	0.0011				
Date 2	Observed	103 (62.8%)	37 (22.6%)	23 (14%)	1 (0.6%)	0 (0%)	164	61 (37.2%)	22.84	<0.001^*^
	Theoretical	100.57	53.06	9.66	0.71	0.02				
Date 3	Observed	63 (48.1%)	9 (6.9%)	32 (24.4%)	25 (19.1%)	2 (1.5%)	131	68 (51.9%)	78.68	<0.001^*^
	Theoretical	42.12	58.36	26.41	4.08	0.03				
Date 4	Observed	73 (68.2%)	10 (9.3%)	18 (16.8%)	6 (5.6%)	0 (0%)	107	34 (32%)	49.18	<0.001^*^
	Theoretical	58.62	39.19	8.52	0.65	0.011				
Date 5	Observed	66 (80.5%)	14 (17.1%)	2 (2.4%)	0 (0%)	0 (0%)	82	16 (20%)	0.005	0.94 ns
	Theoretical	65.72	15.05	1.19	0.04	0.0005				

*Denotes statistical differences and ns denotes non-statistical differences based on the *t-*test on proportion analyses at *p* < 0.05.

When the competition intensity increased across the sampling dates, the number of ovipositions per aphid was higher. In this regard, the proportion of parasitized aphids during the mid-season also increased, reaching its maximum on date three (51.9% of parasitized aphids). On date three, multiparasitism was very frequent, reaching a maximum of the multiparasitism rate of 24.4% (by two parasitoid species) and a minimum of the single-parasitism rate (6.9%). Consequently this date coincides with a five fold mean reduction in aphid densities from the start of the experiment Fig. 1 (44.25 ± 12.40 to 8.7 ± 1.43) and the number of unparasitized aphids was higher than expected (Table 1).

### Proportions of the main parasitoid species

**Figure 4:**
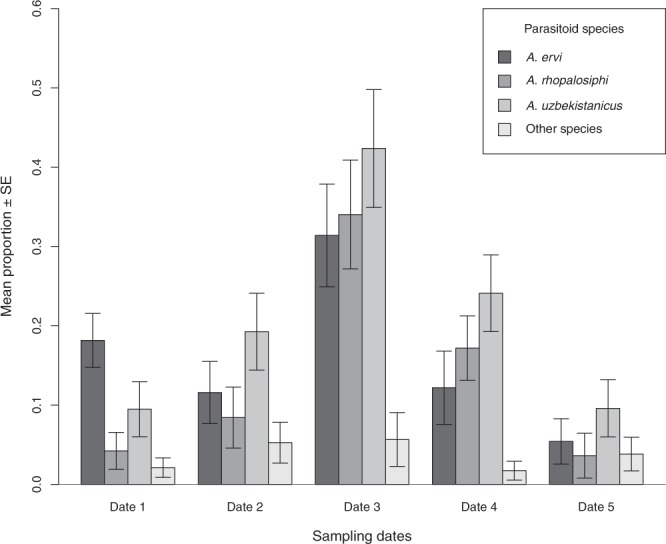
Mean proportion of detected parasitoid species (±standard error (SE)) per field per each sampling date by the molecular approach. Different bars represent different parasitoid species. Proportions per parasitoid species were calculated on the total number of aphids sampled per field and per date. In the screened samples, each aphid could be parasitized by one to four different parasitoid species (see Table 1 for details).

**Figure 5:**
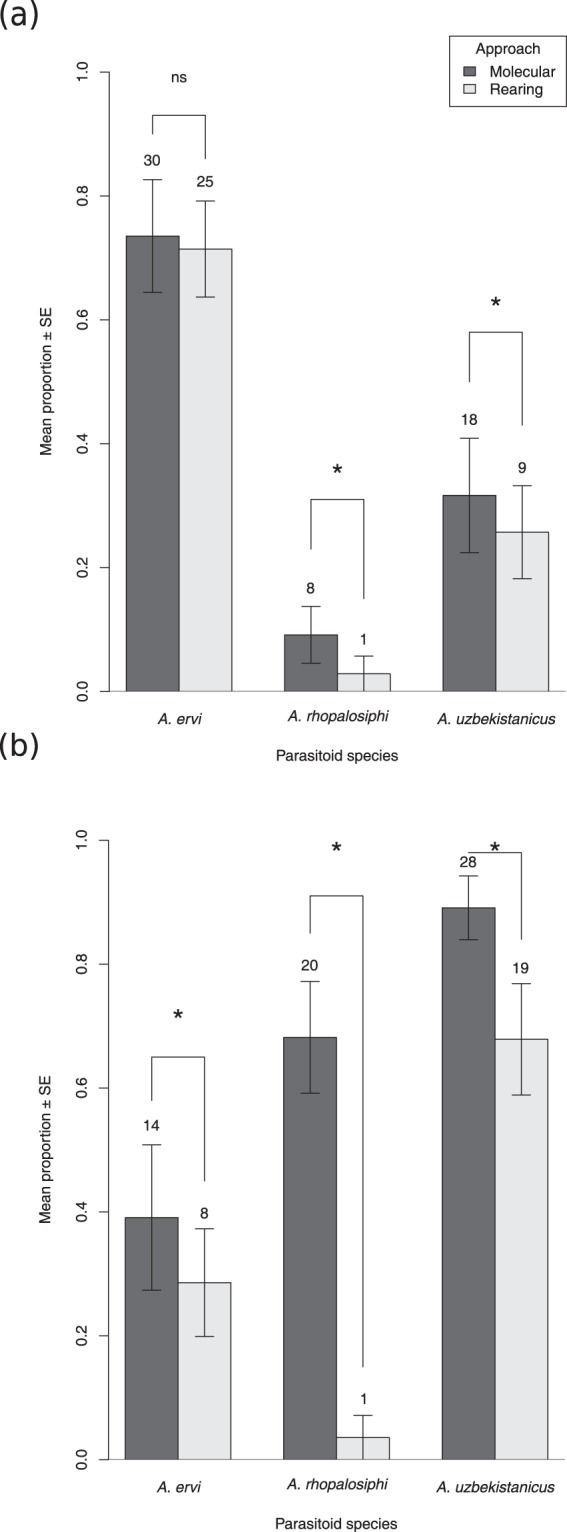
Mean relative proportions of each detected parasitoid species inside parasitized aphids (by molecular approach) and the mean number of adult emergences (by rearing approach) of the three most abundant parasitoid species (±standard error (SE)) in the case of: (**a**) high aphid abundance (date one); and (**b**) low aphid abundance (date four). For the molecular approach, the numbers on each column represent the minimal number of ovipositions for each parasitoid species (there could be more than one parasitoid species in one aphid). For the rearing approach, the numbers on each column represent the number of emerged individuals of each species (morphological identification). * denotes statistical differences and ns denotes non-statistical differences based on the *t-*test on proportion analyses at *p* < 0.05.

The most frequent parasitoid species detected by the molecular approach during all five sampling dates were: *Aphidius uzbekistanicus* (147 ovipositions), *A*. *ervi* (111 ovipositions) and *A*. *rhopalosiphi* (92 ovipositions), the total DNA detection rates per parasitoid species were 59.99% ± 4.88; 47.86% ± 5.11 and 37.40% ± 4.91, respectively. Other parasitoids represented only 39 of the 389 ovipositions (~10%) during the five sampling dates (Fig. 4). During the two contrasting dates of the aphid population growth, 517 individuals of *S*. *avenae* were analysed using the rearing approach. A total of 77 adult parasitoids emerged, with 2 *A*. *rhopalosiphi*, 33 *A*. *ervi* and 28 *A*. *uzbekistanicus*, the proportion and abundance of each parasitoid species per sampling date are provided in Fig. 5.

When the aphid abundance was high (date one), the mean proportion of detected/emerged parasitoid species for both approaches (molecular and rearing respectively) was significantly lower for *A*. *rhopalosiphi* (*t* = 4.162, *p* < 0.001). Similar results were found for *A*. *uzbekistanicus* (*t* = 2.561, *p* = 0.005), whereas for *A*. *ervi* there was no difference (*t* = 1.197, *p* = 0.115). During the sampling date when the aphid abundance was low (date four), all parasitoid species showed a significant reduction in the proportion of emerging parasitoids relative to the proportion of parasitized hosts estimated by DNA detection. This is particularly interesting for *A*. *rhopalosiphi*, for which a significantly lower proportion of emerged individuals was observed through the rearing approach compared to the proportion of molecularly detected parasitoids in aphids (*t* = 9.569, *p* < 0.001). In the case of the rearing approach for this particular species, nearly no emergences were observed, whereas the molecular detections were similar to the other parasitoid species. Accordingly, for the other two parasitoid species, a significantly lower proportion of emerged individuals was detected through the rearing approach (*t* = 3.693, *p* < 0.001; *t* = 2.489, *p* = 0.007 for *A*. *uzbekistanicus* and *A*. *ervi*, respectively) (Fig. 5).

## Discussion

By detecting parasitoid species in a single aphid with a molecular approach, which has been proved as a reliable methodology to identify parasitism status of aphids^50^, it is possible as well to determine the identity of the competitors at immature stages and estimate the effective ovipositions. By using this approach in combination with the classical rearing, it is possible to study interspecific competition during the final development of the different parasitoid species. Thus, the adult emergence rate can be used as a proxy for the survival of the parasitoid species, when compared to the oviposition detection. On the other hand, using the number of detections as a proxy for effective ovipositions provided us with an estimation of the multiparasitism occurring in the system. The latter has been observed to be particularly frequent in periods of low aphid density, when fewer potential resources are available.

### Multiparasitism frequency in the field and aphid abundance

Our results sustain our first hypothesis in terms of a higher multiparasitism rate in periods of low host resources (higher competition), with patches with low aphid densities, which could potentially aggregate different parasitoid species in the middle and late part of the season, confirming data from previous studies^36,37^. Indeed, we observed that the aphid density decreased over the sampling season. This decrease in available resources may have increased competitive interactions among parasitoid species. As the number of potentially exploitable hosts is reduced, female parasitoids have fewer opportunities to lay eggs, increasing the potential number of parasitoid species that could oviposit into a single aphid (multiparasitism rate and total parasitism rate). Therefore, the observed augmentation of multiparasitism and number of ovipositions (with a maximum at the middle of the season, Fig. 2) could be a response to this lack of host resources for egg laying.

### Aphidius species’ competitive abilities in response to resource availability

The reduction in aphid density from the mid-late period may have affected the parasitism rates for each of the three main parasitoid species studied, when higher parasitism rates were observed. This could mean that the efficiency of host exploitation by parasitoids is higher in terms of locating and parasitizing hosts when less aphids are present in the system. This study found higher abundances of the best competitor (*Aphidius ervi)* when aphid resources were scarce. The survival of the inferior competitor (or with less competitive abilities) decreased in host patches when fewer resources were available in the field. Here, we show that the inferior competitor (*A*. *rhopalosiphi*) was intensively affected at low aphid densities, as we observed a high number of ovipositions (molecular detection), but very few surviving adult emergences, despite some aphids remaining unparasitized, supporting the hypothesis that the best competitor (*A*. *ervi*) is much more successful than *A*. *rhopalosiphi* and lays eggs on hosts already parasitized by the inferior competitor.

Interestingly, the overall prevalence of *A*. *ervi* was the highest in this system (in line with previously reported results in^33^) and although *A*. *rhopalosiphi* and *A*. *uzbekistanicus* detection rates were higher in the mid-late period, *A*. *ervi* was still the most successful parasitoid species with more adult emergences. Nevertheless, in Western Europe, *A*. *rhopalosiphi* is considered to be the most abundant cereal aphid parasitoid species, as it is present in the field over the entire year in high abundance, even when other parasitoid species start to forage on cereal aphids^35^. In the Chilean cereal fields, the most abundant species, *A*. *ervi*, may trigger more defensive responses in aphids (e.g., cornicular secretions) making them less likely to be parasitized by *A*. *rhopalosiphi*. Recent studies have also highlighted the role of resource distribution, showing that *A*. *rhopalosiphi* prefers more aggregated host patches^51^. Therefore, in a system with a high prevalence of *A*. *ervi*, which is known to be a patch disturber (aggressive behaviours of patch exploitation)^52^, aphids may be less aggregated, affecting patch encounters by *A*. *rhopalosiphi*.

Alternatively, the role of other general predators, such as coccinellids and carabid beetles, could limit total resources for parasitoids, indirectly affecting the interactions among parasitoids^53^. Chilean population densities of aphids are lower than those observed in Western Europe and the role of generalist predators in the control of cereal in aphids in Chile is well documented^36,54^.

### Host discrimination and single versus multiple ovipositions

We also present data regarding some interspecific host recognition among the main *Aphidius* parasitoid species in the wheat fields of Central Chile, as the minimal observed number of ovipositions of these parasitoids (all species together over the whole sampling period) differed significantly from the expected random host acceptance distribution. In particular, more unparasitized hosts than expected were observed. These results suggested that aphid parasitoids were attacking already parasitized hosts more often than expected (even in cases where unparasitized hosts were available) as single species parasitization events were less frequent than expected. This may indicate that at least some of the parasitoid species foraged on already parasitized hosts in the field, either because these species do not have host discrimination abilities, or they could ignore these cues as they detect these hosts more efficiently. Moreover, particularly in periods of host scarcity, even if this ability to recognize already parasitized hosts is beneficial for some parasitoid species, this could also be omitted by parasitoid females accepting those previously parasitized aphids, especially for superior competitors^22^. However, when comparing the competition intensity at two contrasting aphid densities and the species survival, there were clear differences between the parasitoid species. Regarding host discrimination, it has previously been experimentally proved that *A*. *ervi* and *A*. *rhopalosiphi* can recognize hosts already parasitized by the other species — through antennal perception (external cues) or after the insertion of the ovipositor (internal cues) — and will avoid parasitizing them^33^. This is particularly true for *A*. *ervi*, which is considered the superior competitor in comparison to *A*. *rhopalosiphi* and *A*. *uzbekistanicus*^31,35^. It is therefore likely that these species were ignoring interspecific parasitism cues. In solitary parasitoids such as *Aphidius*, a parasitized host is considered to be a lower quality resource in comparison with unparasitized hosts and should then be avoided, as a strategy for partitioning the host resource between competitors. However, in a scenario of a low abundance of host resources, as we have observed here, the competition between parasitoid species could increase and thus multiparasitism would be more frequent. Similarly, at the parasitoid guild level, it has been suggested that foraging strategies in unfavourable conditions may oblige female parasitoids to forage opportunistically, accepting less suitable hosts^55^. Parasitoids with a greater host range, such as *A*. *ervi*, could increase their efficiency of finding hosts by choosing aphids already parasitized by more specialist parasitoids such as *A*. *rhopalosiphi* (easier to recognize). This has already been suggested, as *A*. *rhopalosiphi* oviposits into more hosts throughout the season, with its high egg production and by only laying one egg per host^35^. So, patches with alarmed hosts could be quickly abandoned by *A*. *rhopalosiphi* and readily used by *A*. *ervi*, which in contrast, does not retreat from alarmed patches^31^. Our results are in accordance with behavioural studies suggesting that *A*. *ervi* can outcompete *A*. *rhopalosiphi* within the host through shorter larval development and/or by a outcompeting the first larval instars^31^. In terms of their host location abilities, natural enemies and parasitoids in particular can use signals from hosts to find and exploit them^56^. Parasitoids that attack aggregated hosts, such as aphids, have evolved the ability to locate and optimize the exploitation of host patches by using kairomones produced by their hosts. When aphids are attacked, they are able to express defensive behaviours, such as the exudation of small waxy droplets containing an alarm pheromone from their cornicles, alerting the aphid colony to disperse^56,57^. This droplet can disturb the female parasitoid, inducing a loss of their oviposition opportunity^58^. Some *Aphidius* parasitoids have the ability to use this alarm pheromone^58–60^, such as *A*. *ervi*, to find host patches. As such, alarmed aphid colonies could be easily detected and alarm pheromones may become especially useful cues at low aphid densities^55^.

We have not studied here the effect of superparasitism, as the methods used here do not allow detecting if several parasitoids eggs/larva of the same species were present inside one aphid. Theory suggest that intraspecific competition will exceed interspecific competition when two species are competing, however, it has been shown that when there is asymmetric competition with many species in a community, then interspecific competition should play a stronger role^61^.

Previously Le Lann and coauthors^31^ have shown that *A*. *ervi* is a superior competitor against *A*. *rhopalosiphi*, even though in this study females were not allowed to lay more than one egg. If females lay more than one egg, as has been suggested for *A*. *ervi*, interspecific competition would be stronger. Laying two eggs could be both a strategy to avoid intra and interspecific competition, as by having more eggs there would be more circulating teratocytes which could stimulate growth for the surviving larvae^62^.

## Conclusions

The results here provide evidence of niche overlapping among these three parasitoid species co-occurring in the system (*A*. *ervi*, *A*. *uzbekistanicus*, and *A*. *rhopalosiphi*). However, the data presented here are limited to one season and country, it is the first to study the temporal multiparasitism of aphids in a real field setting. At least for the habitat generalist *A*. *ervi* and the habitat specialist *A*. *uzbekistanicus*, this could alter the survival of these competitors by promoting the interaction of different strategies in the agroecosystem. This also has been suggested for other *Aphidius* species exploiting the same host, supporting a partial niche partitioning in times of scarce aphid resources, shaping the assemblage under high competition. This effect could be contributing to the coexistence of parasitoid species, at least in the main period of aphid population growth^15^.

Regarding the biological control of aphid pests, this research suggests that in periods of resource scarcity, negative interactions such as interspecific competition are likely to occur, which may produce redundancies in biological control and result in lower pest regulation. Despite this, previous studies have suggested that the biological control of cereal aphids in Central Chile has an important effect on pest suppression^36,37^, even when some negative interactions, such as competition for oviposition, are occurring. Nevertheless, this study did not take into account the interactions and use of other less abundant aphids (such as *Rhopalosiphum padi* Linnaeus and *Metopolophium dirhodum* Walker) which are also host resources for this parasitoid assemblage. In this sense, interspecific competition among parasitoid species could also be altered by the presence and abundance of these other hosts.

Other aspects, such as the vulnerability of these species to other biotic mortality pressures (fungi, predators, hyperparasitoids) should also be considered in future studies, as parasitoids cannot remove the resources after their exploitation (oviposition), as in the case of predators (consumption).

## Supplementary information

R script: newcalc.R
