# Supplementary material for: Interspecific competition among aphid parasitoids: molecular approaches reveal preferential exploitation of parasitized hosts
Source: Sci Rep. 2019 Dec 23;9:19641. doi: 10.1038/s41598-019-56187-3 (PMC6928038; doi:10.1038/s41598-019-56187-3)
Supplement: Supplementary file 1 — R script: newcalc.R [file 41598_2019_56187_MOESM1_ESM.pdf]

Article Title: Interspecific competition among aphid parasitoids: molecular approaches reveal preferential exploitation of parasitized hosts.

Authors: Sebastián Ortiz-Martínez[1], Jean-Sébastien Pierre[2], Joan van Baaren[2], Cécile Le Lann[2], Francisca Zepeda-Paulo[1] and Blas Lavandero[1]*

Affiliation and addresses of the authors:

[1] Universidad de Talca, Instituto de Ciencias Biológicas, Laboratorio de Control Biológico, Avda. Lircay s/n, Talca, Chile.

[2] Université Rennes (Univ Rennes), UMR-CNRS 6553, ECOBIO, F-35042 Rennes, France

eMail: blavandero@utalca.cl

[*] Corresponding author

```
#==============================================================================
#                     newcalc: a novel function to calculate the theoretical
#                     counts of multiple parasitism on the aphids
#==============================================================================
newcalc= function(n,K,nrep=10000)
{
tabres=NULL
for(j in 1:nrep)
{
repart=rep(0,5)
lst=NULL
counts=rep(0,n)
for(i in 1:4)
{
lst[[i]]=sample(subjects,K[i],replace=T)
counts[subjects %in% lst[[i]]]=counts[subjects %in% lst[[i]]]+1
}
for(i in 0:4) repart[i+1]=length(counts[counts==i])
tabres=rbind(tabres,repart)
}

theor=apply(tabres,2,sum)/nrep
cat("\nObserved and theoretical counts without
grouping\n===============================================\n")
print(rbind(observed,theor))


# Accumulation des effectifs th?oriques inf?rieurs ? 5
for (j in 5:1)
{
if(theor[j]<5) { theor[j-1]=theor[j-1]+theor[j]
                observed[j-1]=observed[j-1]+observed[j]} else break()
}
theor=theor[1:j]
observed=observed[1:j]
cat("\nObserved and theoretical counts after
grouping\n=============================================\n")
df=j-1
print(rbind(observed,theor))
Chi=sum((observed-theor)^2/theor);Chi
cat("Chisq = ",Chi," df = ",df,"pval = ",1-pchisq(Chi,df),"\n")
#end function newcalc
}
#==============================================================================
#                     Date 1
#==============================================================================
# Order : uzbe,ervi,rhopa,pici and others
n=157
subjects=1:n
K=c(18,30,8,1)
observed=c(105,41,9,2,0)

newcalc(n,K)
#==============================================================================
#                     Date 2
#==============================================================================
# Order : uzbe,ervi,rhopa,pici and others
n=164
subjects=1:n
K=c(34,22,16,8)
observed=c(103,37,23,1,0)
newcalc(n,K)
#==============================================================================
#                     Date 3
#==============================================================================
# Order : uzbe,ervi,rhopa,pici and others
n=131
subjects=1:n
K=c(60,41,46,1)
observed=c(63,9,32,25,2)
newcalc(n,K)
#==============================================================================
```

```
#                     Date 4
#===============================================================================
# Order : uzbe,ervi,rhopa,pici and others
n=107
subjects=1:n
K=c(28,14,20,2)
observed=c(73,10,18,6,0)
newcalc(n,K)
#===============================================================================
#                     Date 5
#===============================================================================
# Order : uzbe,ervi,rhopa,pici and others
n=82
subjects=1:n
K=c(7,4,2,5)
observed=c(66,14,2,0,0)
newcalc(n,K)
```
